# Passive range of motion in patients with adhesive shoulder capsulitis, an intertester reliability study over eight weeks

**DOI:** 10.1186/s12891-015-0495-4

**Published:** 2015-02-22

**Authors:** Satya Pal Sharma, Anders Bærheim, Alice Kvåle

**Affiliations:** Department of Global Public Health and Primary Care, Research Group for General Practice, University of Bergen, Bergen, Norway; Department of Global Public Health and Primary Care, Physiotherapy Research Group, University of Bergen, Bergen, Norway

**Keywords:** Adhesive capsulitis, Reliability, Passive range of motion, Plurimeter

## Abstract

**Background:**

Measuring range of motion (ROM) in the shoulder joint is important for the diagnosis and monitoring of change over time. To what degree passive ROM can be trusted as a reliable outcome measure was examined as part of an on-going randomized controlled trial for patients with shoulder capsulitis. The aim of this study was to examine intertester reliability of passive ROM in the shoulder joint over a period of eight weeks in patients with adhesive capsulitis stage II.

**Methods:**

Fifty patients with a clinical diagnosis of adhesive shoulder capsulitis were examined by two independent testers. A predefined protocol was used for measuring passive range of motion with an inclinometer, a plurimeter, in both affected and non-affected shoulders three times; at the start of the study and after 4 and 8 weeks.

**Results:**

Very good to excellent intertester agreements were found for most parameters for the affected arm at all three test points. The intraclass correlation coefficient (ICC 2.1) values ranged from 0.76 to 0.98, i.e. from very reliable to excellent. The measurement error was in general small for the affected arm (5°–7°). ICCs were slightly lower for the non-affected arm at 8 weeks, but with acceptable measurement errors.

**Conclusions:**

Intertester reliability between two testers was very good at three visits over a time period of eight weeks using a plurimeter to measure passive range of motion in patients with adhesive shoulder capsulitis. This method can reliably determine passive range of motion in this patient population and be a reliable outcome measure.

## Background

Range of motion (ROM) in the shoulder joint is among the commonly used clinical criteria for diagnostic purposes and to monitor effectiveness of given treatment [1]. ROM is often used as an outcome measure in studies observing effect of intervention in stiff joints and in shoulder pain [2-4]. Therefore it is important that the movement measured is reproducible without much variation, independent of instrument being used.

Shoulder capsulitis is a painful condition affecting between 2 - 5% of the adult population [5-7]. There is a global reduction of active and passive movement, generally in a capsular pattern characterized by most reduction of external rotation, less of abduction and least of internal rotation. Reliable measurement of ROM is therefore essential for the correct diagnosis of adhesive capsulitis of the shoulder, as this is mainly a clinical diagnosis. Measurement variations in patients with shoulder capsulitis are bound to occur due to pain, fear of pain, stiffness, fatigue and measurement error at any one given time point [1]. To our knowledge no intertester reliability study has been conducted in this patient group. Reliability of measurements is therefore essential on both the affected and the non-affected side for diagnostic purposes and over time to monitor progression. Earlier intertester reliability studies have usually measured only affected [8-10] or only non-affected shoulders [11,12] and with participant numbers below 35, and ROM has only been measured at one visit or with an insignificant time difference between measurements [8,10-15]. Most former studies have only reported correlation coefficients and have not reported standard error of measurement (s_w_) [8,14,16,17]. Although as many as 50 patients have recently been recommended to be included in reliability studies (p. 126 in de Vet et al., [18]), only a few studies have examined this many participants [8,17,19,20]. See Table 1 for an overview of former studies.

**Table 1:** **Summary of intra- and intertester reliability studies for range of motion on both the affected and the non-affected shoulders using different measuring modalities**

**Authors**	**Study sample & Size (N)**	**Movement**	**Side measured affected/ non-affected**	**Measuring instrument**	**Point estimates or over time**	**Study type**	**ICC**	**Sw**
Pandya et al. [19]	Muscular dystrophy N = 150	PROM: ABD	Affected	Goniometer	Point estimates	Inter-tester & intra-tester	0.67	No
Riddle et al.[8]	Shoulder pain N = 100, two groups of 50 each	PROM: ABD	Affected	Goniometer	Point estimates	Inter-tester & intra-tester	0.87	No
		PROM: ER					0.88	
		PROM: IR					0.55	
Croft et al. [21]	Shoulder complaints N = 6	PROM: EL	Affected	Protractor	Point estimates	Inter-tester	0.95	No
		PROM: ER	Affected				0.43	
Green et al.[16]	Shoulder pain & stiffness N = 6	AROM: FLEX, ABD, ER, IR, HBB	Affected	Plurimeter	Point estimates	Inter-tester & intra-tester	0.62–0.95	No
Sabari et al. [14]	Rehab.patients N = 11	AROM: FLEX, ABD	Mixed	Goniometer	Point estimates	Intra-tester	0.73–0.78	No
	Normal N = 19	PROM: FLEX, ABD					0.78–0.81	
MacDermid et al. [9]	Shoulder pathology	PROM: ER	Affected	Goniometer	Point estimates	Inter-tester & intra-tester	0.85 & 0.80	Yes
	N = 34							
Hayes et al. [10]	Shoulder pathology	AROM: ABD	Affected	Goniometer, visual est., photography	Point estimates	Inter-tester & intra-tester	ICC(Rho) 0.69	Yes
	N = 8							
		AROM:ER	Affected	Goniometer			0.64	
		AROM: HBB		Measuring tape			0.39	
Hoving et al. [22]	Shoulder pain & stiffness N = 6	AROM: ABD	Affected	Plurimeter	Point estimates	Inter-tester & intra-tester	0.51	No
		ER in neutral					0.29	
		ER in abd					0.11	
		IR in abd					0.06	
		HBB					0.80	
Awan et al. [23]	Normal (athletes) N = 56	ER without scapular stabilization	Unaffected, right & left side	Digital inclinometer	Point estimates	Inter-tester/ intra-tester	0.41–0.51	No
		IR standard technique					0.50–0.66	
		IR with scap.stab.						
		IR without scap. stab						
de Winter et al. [17]	Shoulder complaints N = 155	PROM: ABD	Affected/ non-affected	Digital inclinometer	Point estimates	Inter-tester	0.83/0.28	No
		PROM: ER					0.90/0.56	
Terwee et al. [20]	Shoulder complaints N = 201	PROM: ABD	Affected/ non-affected	Visual estimation	Point estimates	Inter-tester	0.67/0.15	Yes
		AROM: EL					0.88/0.76	
		PROM: ER					0.73/0.34	
Nadeau et al. [12]	Normal N = 30	AROM: ELEV, PROTR, RETRAC	Non-affected	Goniometer & tape measure	Point estimates	Inter-tester & intra-tester	0.78–0.46	No
							0.75–0.32	
Tveitå et al. [15]	Shoulder adhesive capsulitis N = 32	PROM: ABD	Affected/non-affected	Digital inclinometer	Point estimates	Intra-tester	0.72/0.89	Yes
		FLEX					0.76/0.61	
		IR					0.81/0.88	
		ER					0.91/0.86	
Mullaney et al. [13]	Shoulder pain N = 20	AROM: FLEX	Affected/non-affected	Goniometer/ digital level	Point estimates	Inter-tester & intra-tester	0.93/0.74, 0.92/0.79, 0.82/0.62	Yes
		ER						
		IR						
Kolber et al. [11]	Normal N = 30	AROM: ABD	Non-affected	Inclinometer	Point estimates	Inter-tester & intra-tester	0.95	Yes
		AROM: ER	Non-affected				0.88	
		AROM: IR					0.93	
De Jong et al. [24]	Hemiplegic shoulder N = 43	PROM: ABD	Affected	Hydro-goniometer	Over time 4, 8 & 20 weeks	Inter-tester	0.97	Yes
		PROM: ER	Affected				0.94	

ABD = abduction, ER = external rotation, IR = internal rotation, HBB = hand behind back, EL = elevation, PROM = Passive range of motion, AROM = Active range of motion, Sw = standard error of measurement, scap. stab = scapular stabilization.

With few exceptions only the inter-tester ICC values are written in the table.

To what degree ROM can be trusted as a reliable outcome measure was examined as part of an on-going randomized controlled trial for patients with adhesive capsulitis of the shoulder. The aims of the study were:

- To determine the reliability of shoulder passive ROM (PROM) bilaterally between two testers in a large number of participants with adhesive capsulitis using a validated measuring instrument, the plurimeter [16,25], over an eight week period.
- To determine if the measurement error remained the same during the eight week period, measured three times four weeks apart.

The intertester reliability was evaluated for PROM in abduction, external rotation, internal rotation and “hand behind back” in patients with shoulder capsulitis.

## Methods

PROM in the shoulder joint is defined as to the extent an investigator can move the arm until pain or stiffness limits the movement. We measured PROM on both the affected side and the non-affected side. There are no set rules regarding measurement intervals or the number of times measurement should take place. To avoid too much pain provocation we decided to measure each movement only once for each tester. Therefore a total of eight measurements for PROM were carried out for each of the two testers on each patient. No standardized time interval was set between tester 1 and 2, and usually only a few minutes elapsed between the two measurement sessions.

### Participants

Patients potentially eligible for inclusion in the randomized controlled trial for treatment of shoulder capsulitis were referred to a primary care clinic by physicians and physiotherapists in the period 2010–2012. The study was approved by the Regional Ethical Committee REK NORD, reference 148/2008, in compliance with the Helsinki Declaration. Informed consent was obtained from all participants, and from the tester appearing in the photographs.

The measurement took place on the second consultation and is hereafter referred to as visit 1 in the study. The PROM testing took place on visit 1, four weeks later (visit 2) and then at eight weeks (visit 3).

To be included in the study patients had to be above 18 years of age, should be able to understand and speak Norwegian, and there should be no contraindications for use of corticosteroids. Participants should have reduced range of motion in a capsular pattern with a reduction of more than 30% of two out of three shoulder movements and none of the three movements (Abduction = ABD, External rotation = ER and Internal rotation = IR) should be normal. All patients were having both pain and stiffness, and can be referred to as being in stage 1 and/or stage 2 [25,26]. Patients with diabetes, asthma, pregnant women and breast feeding mothers were excluded from the study.

The first 50 participants recruited in the main study were included in the intertester reliability study, comprising 22 men and 28 women, age ranging from 38 years to 75 years (mean age 52 years; SD 9.3). Along with other demographic data, information regarding the affected shoulder, such as the side affected, how long the condition had lasted and details of any previous treatment, was collected before taking the PROM measurements. Mean pain intensity measured with Numerical Pain rating Scale (NPRS) was 6.8 (SD 1.7) and Shoulder Pain and Disability Index (SPADI) was 63.0 (SD 19.3). Four of the included participants had bilateral capsulitis. In these patients we chose the more affected side as the “affected” and the other side as the “non-affected” side.

### Testers

Both testers were experienced general practitioners and had experience with measurements of shoulder movements with goniometer from a former pilot study. They had also trained with plurimeter on each other and on patients with shoulder capsulitis before the start of the study. Tester 2 (SS), who is also a physiotherapist, had experience in measurements of ROM. The testers planned beforehand how the measurements were to be carried out and standardised the procedures. The two testers performed ROM-testing in the same order; tester 2 always tested first and tester 1 last. The two testers kept their measurements records confidential and inaccessible to each other throughout the duration of the study until all the data was collected.

### Measurements

**Figure 1:**
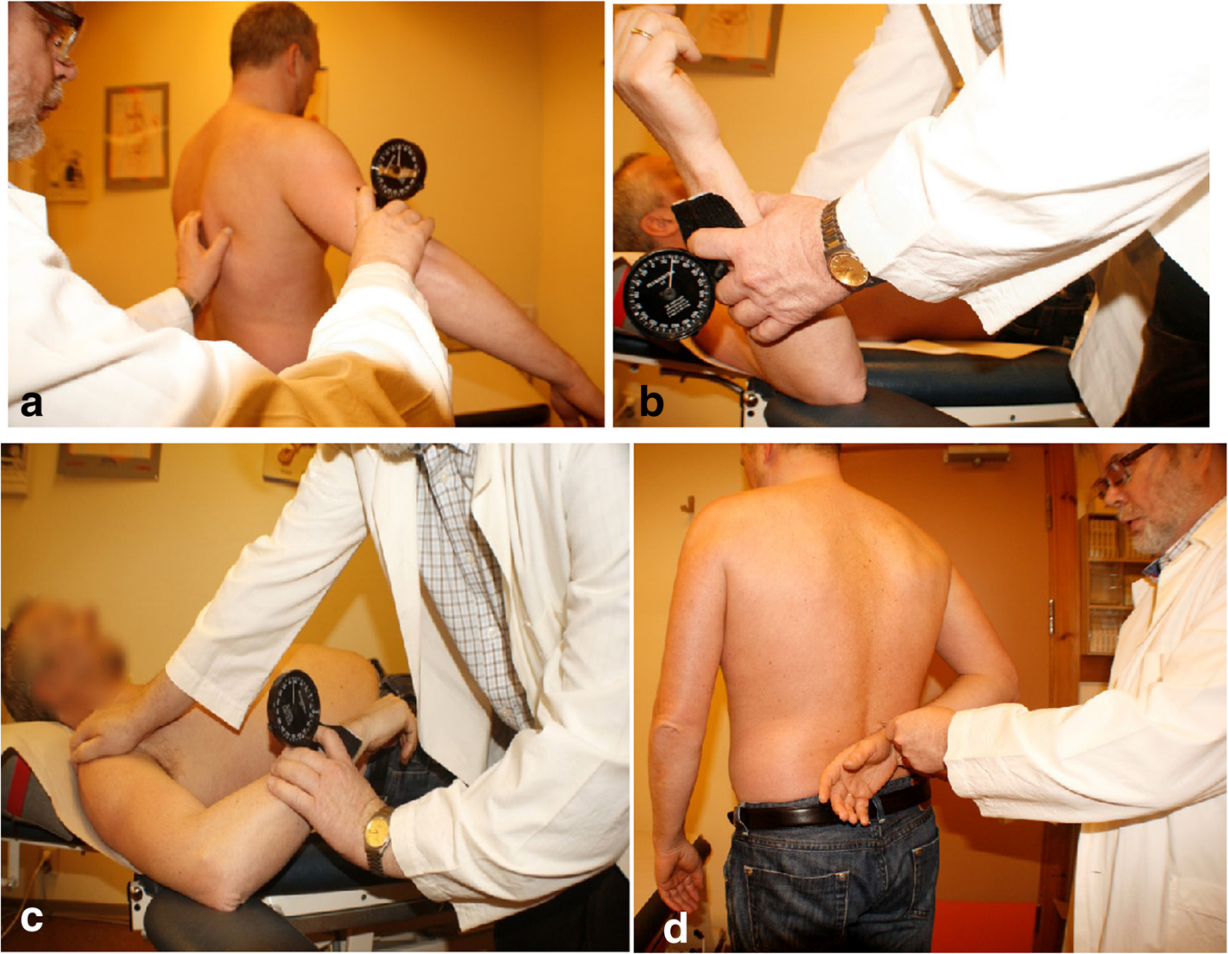
**Measurement of passive range of motion in shoulder. a)** Abduction. **b)** External rotation. **c)** Internal rotation. **d)** Hand behind back.

The Plurimeter-V gravity inclinometer (Plurimeter-V inclinometer; Dr. Rippstein, Zurich, Switzerland) was used in this study to measure PROM for abduction, external rotation and internal rotation. A plurimeter is a hand-held instrument for measuring relative angles between surfaces. In the gravity referenced inclinometer the starting position for the measurement is fixed, which is either 0° or 180°. The reliability of this instrument for measuring shoulder and scapular passive range of motion (PROM) has been examined in previous studies [16,27]. The exact technique of measurement was standardised considering the position of the patient, position of the arm in relation to the body, and position of the plurimeter in relation to the arm (Figure 1 a-d). The starting position of the plurimeter was from 0 for every measurement with the instrument on the arm prior to the start of the shoulder movement. This minimizes placement error. To determine the end point of range, the arm was passively moved up to the tolerance level of pain or when it was not possible to move it further due to stiffness.

The following individual passive movements were measured:

#### In standing

##### Passive gleno-humeral abduction (ABD)

The patient was in standing position and the tester stood partly behind and partly to the side of the patient to be measured. The scapula was stabilized by the tester holding the inferior angle of the scapula between thumb and index finger of one hand and holding the patients arm just proximal to the patient’s elbow while at the same time holding the plurimeter between 2nd and 3rd finger on the dorsal aspect of the upper arm. Care was taken to hold the plurimeter base in a straight line on the upper arm. The arm was then passively abducted. The end point was reached either when the pain was reported as unbearable by the patient or the scapula began to rotate and the examiner could not hold the scapula in place. The reading on the plurimeter was then registered.

##### Hand behind back (HBB)

HBB was measured in centimetres. The patient was in standing position and the distance was measured in centimetres (cm) by placing the patient’s hand behind the back as far as it could reach within pain limits with the ventral side of palm facing outwards. The end point was considered to be the highest landmark reached with the upper end of the radius proximal to the wrist. We chose the distal end of radius as the highest landmark to avoid measurement errors involving movement of wrist and thumb. The starting point (0 point) was taken from the posterior inferior iliac spine (PIIS). If the hand did not reach PIIS, the distance to PIIS was denoted in minus centimeters (−cm). In case the hand did not reach medially enough, parallel lines were drawn from the 0 point and the distance between them was measured horizontally. Though a complex movement, this is a pragmatic way of measuring internal rotation of the shoulder joint and is commonly used in clinical situations [16]. Studies have however demonstrated that HBB does not measure the exact range of internal rotation [28,29].

#### In supine lying

##### Passive external rotation (ER) in 45° of abduction

The patient was lying supine with about 45° of gleno-humeral abduction and the elbow was kept at 90° of flexion and the forearm was kept in mid position. The plurimeter was placed between the shaft of radius and ulna in a straight line. The arm was rotated in external rotation. If the arm did not reach 0°, the ROM was noted in minus degrees.

##### Passive internal rotation (IR) in 45° of abduction

The position of the patient was the same as for measuring ER. The arm was then rotated in internal rotation. The reading on plurimeter was registered at the end point of movement i.e. when the pain was unbearable or the arm could not be moved further.

### Statistics

Descriptive statistics with mean measurement including standard deviation (SD) for each movement is presented. Reliability refers to relative agreement as well as absolute measurement error. For calculation of reliability the Intraclass correlation coefficients (ICC model 2.1) was used, as this accounts for both systematic and random error. ICC is a reliability parameter and ranges between 0 and 1, and there are no fixed standards regarding what can be considered as acceptable. According to Eliasziw and co-workers [30], ICC values from 0.60 to 0.79 indicate moderate reliability, values ≥0.80 to 0.90 as very reliable, and >0.90 as excellent. For absolute agreement, which is the actual difference in measurements (i.e. absolute measurement error in degrees and centimeters), the size of measurement error was calculated. Bland and Altman [31] have suggested estimating within-subject standard deviation (s_w_), i.e. the common SD of repeated measurements, derived from one-way analysis of variance. Statistical analysis was carried out using the IBM SPSS Statistics version 19, software program.

## Results

Mean raw data for measurement of abduction, external and internal rotation and hand behind back are listed for both the affected and the non-affected arm in Tables 2, 3, 4 and 5.

**Table 2:** **Mean range and standard deviation (SD) for two testers for abduction (ABD) in 50 patients with shoulder capsulitis**

**Visit**	**Tests**	**ROM tester 1**	**ROM tester 2**	**ICC**	**s**_**w**_
		**ROM° (SD)**	**ROM° (SD)**	**2,1**	**°**
1.	ABD non-affected	87.3 (7.7)	88.4 (6.6)	0.91	2.1
	ABD affected	53.2 (17.0)	54.1 (15.1)	0.83	6.7
2.	ABD non-affected	88.3 (6.8)	89.0 (4.7)	0.88	2.0
	ABD affected	60.6 (17.9)	61.7 (18.7)	0.90	5.9
3.	ABD non-affected	89.7 (2.9)	89.7 (1.6)	0.64	1.4
	ABD affected	65.4 (19.5)	68.3 (19.5)	0.86	7.2

Relative agreement is reported with intraclass correlation coefficient, ICC (2.1), and absolute measurement error is reported with within-subject standard deviation, s_w,_ between the two testers. ROM = Range of motion.

**Table 3:** **Mean range and standard deviation (SD) for two testers for external rotation (ER) in 50 patients with shoulder capsulitis**

**Visit**	**Tests**	**ROM Tester 1**	**ROM Tester 2**	**ICC**	**s**_**w**_
		**ROM° (SD)**	**ROM° (SD)**	**2,1**	**°**
1.	ER non-affected	70.8 (16.0)	75.0 (17.9)	0.83	6.5
	ER affected	18.6 (16.2)	22.1 (16.1)	0.90	4.5
2.	ER non-affected	71.8 (14.1)	76.4 (15.7)	0.80	6.0
	ER affected	25.0 (19.2)	28.6 (17.6)	0.89	5.8
3.	ER non-affected	72.0 (13.2)	78.5 (11.3)	0.69	5.7
	ER affected	34.0 (22.6)	35.3 (19.2)	0.91	6.2

**Table 4:** **Mean range and standard deviation (SD) for two testers for internal rotation (IR) and hand behind back (HBB) in 50 patients with shoulder capsulitis**

**Visit**	**Tests**	**ROM Tester 1**	**ROM Tester 2**	**ICC**	**s**_**w**_
		**ROM° (SD)**	**ROM° (SD)**	**2,1**	**°**
1.	IR non-affected	73.0 (14.3)	77.9 (13.9)	0.76	6.2
	IR affected	40.7 (14.9)	42.4 (14.1)	0.85	7
2.	IR non-affected	72.9 (13.0)	78.0 (13.4)	0.78	5.4
	IR affected	44.3 (16.9)	47.3 (17.8)	0.85	6.4
3.	IR non-affected	73.8 (10.8)	80.3 (9.9)	0.63	5.1
	IR affected	51.0 (18.3)	54.1 (18.0)	0.89	5.6

**Table 5:** **Mean range and standard deviation (SD) for two testers for hand behind back (HBB) in 50 patients with shoulder capsulitis**

**Visit**	**Tests**	**ROM Tester 1**	**ROM Tester 2**	**ICC**	**s**_**w**_
		**ROM (SD)**	**ROM (SD)**	**2,1**	**cm**
1.	HBB non-affected (cm)	18.3 (5.7)	18.4 (5.8)	0.97	2.1
	HBB affected (cm)	0.2 (6.9)	1.5 (7.0)	0.91	1.9
2.	HBB non-affected (cm)	19.0 (5.8)	19.0 (5.5)	0.98	0.9
	HBB affected (cm)	4.9 (7.3)	4.3 (7.9)	0.94	1.8
3.	HBB non-affected (cm)	19.1 (5.0)	19.4 (5.3)	0.96	1.1
	HBB affected (cm)	7.9 (8.6)	8.7 (7.9)	0.96	1.6

### Abduction (ABD)

Very good to excellent reliability calculated with ICC 2.1 was found during all three visits, except for the 3rd visit for the normal side (Table 2).

The measurement error (s_w)_ for the affected arm ranged from 6.7° at first visit, 5.9° at the 2nd and 7.2° at the last visit, whereas s_w_ for the non-affected arm was between 1.4 and 2.1°.

### External rotation (ER)

Very good to excellent reliability was found at all three visits, except for a moderate reliability shown for the non-affected arm on the third visit (Table 3).

The measurement error (s_w_) for the affected arm ranged between 4.5°, 5.8° and 6.2°. The s_w_ was almost the same for the healthy arm: 6.5° on the first visit and 5.7° on the last.

### Internal rotation (IR)

Good reliability calculated with ICCs was found on both the normal and affected side for all three visits, except for moderate reliability (ICC 0.63) shown in the last visit for the normal arm. Measurement error for IR ranged from 5.6° to 7.0° on the affected side and from to 5.1° to 6.2 on the healthy side (Table 4).

### Hand behind back (HBB)

Excellent reliability was found on both the normal and the affected side when measuring HBB. The measurement error ranged from 1.6 cm to 1.9 cm on the affected side, and from 1.1 cm to 2.1 cm on the normal side (Table 5).

**Figure 2:**
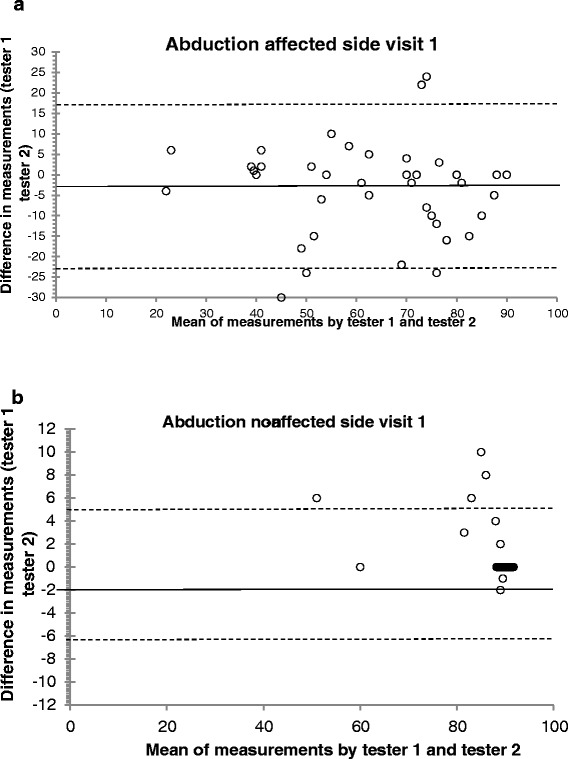
**Bland-Altman plot with mean abduction values for a) affected and b) non-affected arm in 50 patients with adhesive capsulitis at visit 1 for both testers plotted against the difference between testers.** For the non-affected arm equal values are dispersed by a factor of 0.1 degrees at 90 degrees.

Graphic scatter plots showed that there were a few outliers (see Figure 2a and b) where the two testers had measured a difference in range of 15° - 20° in a couple of patients.

## Discussion

This large cohort study demonstrated very good to excellent intertester reliability when examining PROM in patients with shoulder adhesive capsulitis stage II. The results in our study are comparable or better than other reliability studies measuring shoulder ROM in normal individuals or in other shoulder populations (Table 1) [8,13,16,17,20,22,24]. To our knowledge this is the first reliability study that has measured passive ROM in patients with adhesive shoulder capsulitis using a plurimeter, whereas most former studies have used a variety of measuring instruments and techniques. The intertester reliability remained excellent at all three visits for examination of the affected side. The unaffected arm had stable measurements over time, while the affected arm changed over time, possibly due to treatment and/or general improvement. The measurement errors were found to vary between ~5°- 7° on all three visits when examining the affected shoulder for passive abduction, and bilaterally when examining external and internal rotation. The measurement error was relatively small when examining abduction (ABD) (~1.5°- 2°) in the normal arm and for measuring hand behind back (HBB) in both arms (~1 cm −2 cm) on all three visits. Some of the good results found in our study may be attributed to training of the testers who practiced the procedures on each other and on patients before the start of the study. Better results have been observed due to increased practice earlier [16].

The ICCs values for the affected side were very reliable on all three visits (ICCs ≥ 0.83 - 0.96). The measurements on the non-affected side had slightly lower ICC values than the affected side, but only at the third visit. De Winter et al. [17] had an ICC of 0.28 on the non-affected side and 0.83 on the affected side for ABD, and 0.56 for the non-affected side and 0.90 for the affected side for ER in patients with painful shoulder. Possibly a combination of low spread in scores and low variance has resulted in a low ICC, albeit with a low measurement error as demonstrated in Figure 2 b.

The absolute measurement error found in our study is generally better than the few studies where ROM in the shoulder has been measured. However, no values have formerly been reported on patients with capsulitis. Hayes et al. [10] found standard error of measurement (i.e. s_w_) to range from 14° - 25° for flexion, ABD and IR. Kolber et al. [11] reported small s_w_ ranging from approximately 2° - 4° in ABD, ER and IR among 30 normal participants. In the study by de Winter et al. [17], 155 participants with shoulder pain were examined, and s_w_ ranged from 14° - 20° for ABD and ER. Muir et al. [1] studied a mixed participant group of 17, and the s_w_ ranged from 6° - 9° in flexion, ABD, ER and IR in supine lying. The measurement error indicates that some variation must be expected when using ROM as an outcome measure. In our study the affected arm had about 1/3 to ½ of the ROM as compared to the non-affected arm at the first visit. Smallest detectable change SDD (√2 x1.96 = 2.77 s_w_) is often used to indicate statistical significant change [32]. An s_w_ of ~5°–7° for ER, IR and ABD and an s_w_ of ~2 cm for HBB for the affected side would indicate that statistical change larger than the measurement error in the effect study would have to be 14°- 19°, and ~5.5 cm. In our study, the SDD values for the affected arm were close to statistical significant change above measurement error from the first to the third visit. Range of motion is an important and reliable outcome measure, and a change of ≥15° is necessary to represent a clinically significant change in patients with adhesive capsulitis. Patients with shoulder capsulitis in stage II generally have a large movement reduction and a change of >15° has a positive impact on functionality in activities of daily living. Clinically important change should be defined within a context, and may sometimes be smaller than the SDD [33].

A sample size of 50 participants and measurement of ROM on both the affected and non-affected side constitutes a large sample size for examination of reliability [18]. Inclusion of 50 patients was based on the recommendations made in “Measurement in medicine” [32]. However, since two testers tested both sides three times, a lower sample size would have been sufficient.

Our sample is representative concerning gender (56% female) and age (mean 52 years) for patients with shoulder adhesive capsulitis in stage II [15]. At inclusion, participants in our study were patients with moderate to severe capsulitis. The numerical pain rating scale (NPRS) ranged from 5 to 9, which characterizes moderate to severe pain and may pose problems in measuring ROM. However, the very good to excellent reliability proves otherwise, i.e. measurements were still reliable in patients with moderate to severe painful stiff shoulders corresponding to stage II. The pre-treatment value for pain and function indicated moderate to severe problems (SPADI values varied from 42 to 98, on average 63). The recruited patients had restricted shoulder movement with more than 30% reduction in two of three PROM values and none of the three movements were normal. We chose to only examine passive ABD, ER, IR and HBB as these are the standard movements for diagnosis of shoulder capsulitis and may also be used over time to monitor progression [34,35]. Since pain and stiffness pose particular problems while measuring PROM, for example in finding out the exact end point of movement, measurement of AROM could have been a good supplement. Studies have shown that AROM is more reliable than PROM, probably because the extra pressure from the examiner while measuring PROM may affect the ROM [36,37].

The strength of this study lies in its good power, representativeness of the condition studied and good to excellent results, as well as being the first study that measures intertester reliability in patients with shoulder adhesive capsulitis with plurimeter. Among limitations it may be mentioned that non-randomization of testers may have induced systematic measurement error, as tester 2 may have provoked pain and thus affected the PROM for tester 1. The testers had two criteria, pain and stiffness, for judging the end of movement and this may also have constituted some source of measurement variation, although small. Despite the non-randomised test-procedure our results are very good.

Although tester 2, who always tested before tester 1, had a tendency to measure a larger range for external and internal rotation, and mostly for the non-affected arm, findings in our study show an overall very good to excellent reliability for measuring PROM in patients with this condition. This is an important finding because measuring PROM is the diagnostic test for adhesive shoulder capsulitis. Little difference in intertester reliability occurred for the duration of the study (eight weeks). Although an intra-tester reliability study with short time intervals was not performed, our results indicate that we can trust the measurements from one tester at different visits also in an effect study.

## Conclusion

Intertester reliability between two testers was very good at three visits over a time period of eight weeks using a plurimeter to measure passive range of motion in patients with adhesive shoulder capsulitis. This method can reliably determine passive range of motion in this patient population and be a reliable outcome measure.
